# Metabolic Stability and Metabolite Characterization of Capilliposide B and Capilliposide C by LC–QTRAP–MS/MS

**DOI:** 10.3390/pharmaceutics10040178

**Published:** 2018-10-08

**Authors:** Zhongzhe Cheng, Xing Zhou, Zhifeng Du, Wenyi Li, Bingying Hu, Jingkui Tian, Lin Zhang, Jiangeng Huang, Hongliang Jiang

**Affiliations:** 1 School of Pharmacy, Weifang Medical University, 7166 Baotong West Street, Weifang 261053, Shandong, China; chengzhzh@wfmc.edu.cn; 2 School of Pharmacy, Tongji Medical College, Huazhong University of Science and Technology, 13 Hangkong Road, Wuhan 430030, Hubei, China; M201575255@hust.edu.cn (X.Z.); duzhifeng@hust.edu.cn (Z.D.); wenyilitj@gmail.com (W.L.); huby@zjams.com.cn (B.H.); jianghongliang@hust.edu.cn (H.J.); 3 Department of Biomedical Engineering, Zhejiang University, Hangzhou 310027, Zhejiang, China; tjk@zju.edu.cn (J.T.); zhanglin@zju.edu.cn (L.Z.)

**Keywords:** Capilliposide B, Capilliposide C, metabolic stability, metabolism, LC-MS/MS

## Abstract

Capilliposide B (LC-B) and Capilliposide C (LC-C), two new triterpenoid saponins extracted from *Lysimachia capillipes* Hemsl, exhibit potential anticancer activity both in vitro and in vivo. However, their metabolic process remains unclear. In this study, the metabolic stability of LC-B, LC-C, and Capilliposide A (LC-A, a bioactive metabolite of LC-B and LC-C) was investigated in human, rat, and mouse liver microsomes, respectively. Thereafter, their metabolites were identified and characterized after oral administration in mice. As a result, species difference was found in the metabolic stability of LC-B and LC-C. All three compounds of interest were stable in human and rat liver microsomes, but LC-B and LC-C significantly degraded in mouse liver microsomes. The metabolic instability of LC-B and LC-C was mainly caused by esterolysis. Moreover, 19 metabolites were identified and characterized in mouse biological matrices. LC-B and LC-C mainly underwent deglycosylation and esterolysis, accompanied by dehydration, dehydrogenation, and hydroxylation as minor metabolic reactions. Finally, the metabolic pathway of LC-B and LC-C in mice was proposed. Our results updated the preclinical metabolism and disposition process of LC-B and LC-C, which provided additional information for better understanding efficacy and safety.

## 1. Introduction

*Lysimachia capillipes* Hemsl (Primulaceae) is traditionally used for cold and arthritis in China [1]. Capilliposides, a series of novel oleanane triterpenoid saponins, are the characteristic chemical markers for *Lysimachia capillipes* Hemsl (Primulaceae) [1,2,3]. Capilliposide B (LC-B) and Capilliposide C (LC-C), two representative compounds of them, exhibited significant cytotoxicities against ovarian carcinoma A-2780, prostate cancer PC3, and DU145 cells [2,4]. As herbal supplement, the chemosenstizing effect of the cotreatment of LC-C and oxaliplatin in esophageal squamous carcinoma cells has been reported [5]. Moreover, total saponin extracts of this herb (LCE) showed an antitumor effect in several human xenograft tumor models without obvious toxicity [6,7]. Because of its wide application and potential therapeutic effect, the standardized planting techniques of *Lysimachia capillipes* Hemsl have recently been developed to increase production and guarantee quality [8].

Previously, pharmacokinetics, tissue distribution, and excretion of LC-B and LC-C were systematically evaluated by our group [9,10,11]. Both compounds showed poor bioavailability and low exposure in tissues after oral administration [9]. The recovery of LC-B was less than 10% in rat excreta after intravenous administration, suggesting that LC-B underwent extensive metabolism prior to excretion [11]. Similarly, extensive metabolism of LC-C in rat intestinal microflora was found and the resultant metabolite pool demonstrated a strong anticancer activity [12]. Moreover, the esterolytic metabolite of LC-C, Capilliposide A (LC-A), also exhibited a significant cytotoxicity against HepG2 cells [12]. However, the metabolic process of LC-B and LC-C remains unclear. Thus, a systemic study is required to investigate the metabolism of LC-B and LC-C.

Although we have systematically evaluated the pharmacokinetics, distribution, intestinal microflora metabolism, and excretion of LC-B and LC-C [9,10,11,12], the primary aim of the present study was to study the metabolic stability of LC-B and LC-C in liver microsomes obtained from different species and to clarify their metabolites in vivo. The results will update information concerning preclinical metabolism and the disposition process of LC-B and LC-C.

## 2. Materials and Methods

### 2.1. Chemicals and Reagents

Capilliposide B (LC-B) and LC-C were isolated from *Lysimachia capillipes* Hemsl (Primulaceae) [2]. The purity of both compounds was higher than 98%, measured by an HPLC-ELSD method [9]. LC-A was hydrolyzed from LC-C with a purity of >98%, as described in a previous report [12]. Dioscin (98% purity, Jiangxi University of Traditional Chinese Medicine, Nangchang, China) was used as internal standard (IS). Midazolam and nomifensine (IS) were purchased from the National Institutes for Food and Drug Control (Beijing, China). Nicotinamide adenine dinucleotide phosphate (NADPH) was purchased from Dingguochangsheng Biotechnology Co., Ltd. (Beijing, China). Pooled liver microsomes originating from different species such as rats, mice, and humans were purchased from BD Gentest (Woburn, MA, USA). Acetonitrile and methanol were HPLC grade and provided by Fisher Scientific (Pittsburgh, PA, USA). Formic acid (≥96%) was purchased from Sinopharm Chemistry Reagent Co., Ltd. (Shanghai, China). The OASIS solid phase extraction (SPE) cartridges were obtained from Waters Corp. (Milford, MA, USA).

### 2.2. Metabolic Stability

We incubated LC-A, LC-B, and LC-C separately with 0.5 mg/mL liver microsomes at a final substrate concentration of 1.0 μM. The reaction mixture contained 5.0 mM NADPH in potassium phosphate buffer (0.1 M, pH 7.4). Incubation was performed in triplicate in a final volume of 0.5 mL at 37 °C. After 5 min pre-incubation, NADPH was added to start the reactions. The incubation system in the absence of NADPH was used as a negative control. Midazolam (0.5 μM) was used as a positive control. An aliquot of 0.5 mL of ice cold acetonitrile containing IS (dioscin, 200 ng/mL) was added into 20 μL of samples to terminate the reactions at 0, 5, 15, 30, 45, 60, and 90 min, respectively. For midazolam, metabolic reactions were quenched at 0, 2, 5, 10, and 20 min by adding 0.5 mL acetonitrile containing IS (nomifensine, 70 ng/mL) to 20 μL of corresponding samples. After centrifugation for 10 min at 17,000× *g*, the supernatant (5 μL) was subjected to analysis.

### 2.3. Metabolism in Mice

#### 2.3.1. Animal Experiments

Kunming mice (20 ± 2 g) were purchased from the Laboratory Animal Center of Tongji Medical College, Huazhong University of Science and Technology (HUST, Wuhan, China). The mice were housed in a standard environment with free access to food and water. The experiment protocol (Project ID: HUST-D-201410009) was approved by the Animal Ethics Committee in HUST.

Prior to experiments, blank urinary and fecal samples were harvested from thirty mice (half male and half female). Among them, three male and three female mice were sacrificed to harvest blank plasma. Next, twenty four mice were randomly divided into four groups (half male and female) and were orally administrated LCE prepared in 0.5% carboxy methylcellulose sodium continuously for 9 days (300 mg/kg/day). After dosing, urine and feces were separately collected at 0–12 and 12–24 h. To harvest the blood samples, mice were sequentially dosed on the tenth day and then sacrificed at the predefined time intervals (30 min, 2, 6, and 12 h). After centrifugation for 10 min at 900× *g* at 4 °C, plasma samples were obtained and stored at −80 °C.

#### 2.3.2. Sample Preparation

Five hundred microliter of the pooled plasma sample was treated with 1.5 mL of acetonitrile, followed by 10 min vortex-mixing. Next, the mixtures were centrifuged for 10 min at 17,000× *g* at 4 °C. The resultant supernatant was evaporated to dryness under a stream of nitrogen. The residue was then dissolved by 200 μL of acetonitrile:water (2:8, *v*/*v*).

Urine samples were cleaned up using the previously reported solid phase extraction (SPE) method with OASIS SPE cartridges (60 mg, 30 μm, 3 mL) [11]. In brief, the cartridges were preconditioned with 1 mL of methanol and then 1 mL of water. Next, SPE columns loaded with 0.5 mL of urine sample were washed with water (1 mL) and then eluted, using methanol (1 mL). The eluate was evaporated to dryness under a stream of nitrogen at 40 °C and reconstituted in 200 μL of acetonitrile:water (2:8, *v*/*v*).

The pooled fecal sample (200 mg) was homogenized and extracted with methanol (2.0 mL) using ultrasonication. Then, samples were centrifuged for 10 min at 17,000× *g* under 4 °C. The supernatant was evaporated to dryness under a stream of nitrogen at 40 °C and reconstituted in 200 μL of acetonitrile:water (2:8, *v*/*v*).

#### 2.3.3. LC-MS/MS Conditions

The HPLC system consisted of a DGU-20A3 degasser, an LC-20ADXR solvent delivery system, a CTO-20AC column oven, an SIL-20ACXR autosampler, and a CBM-20A controller from Shimadzu (Kyoto, Japan). The 4500 QTRAP^®^ mass spectrometer (SCIEX, Concord, ON, Canada) equipped with a Turboionspray^®^ source was used. Previously, an assay for simultaneous determination of LC-A, LC-B, and LC-C was established by our group [11]. The chromatographic and MS conditions in the present study were the same as those in our previous report [11]. Briefly, the separation was achieved on an Aquasil C18 column (50 × 2.1 mm, 5 μm, Thermo Electron, Bellefont, PA, USA). Mobile Phase A was water containing 0.1% formic acid, and Mobile Phase B was methanol. The flow rate was 0.8 mL/min. The chromatographic gradient condition was as follows: 0–0.2 min 10% B, 0.2–1.5 min 10 to 90% B, 1.5–2.2 min 90% B, 2.2–2.3 min 90 to 10% B, and 2.3–3.0 min 10% B. Midazolam was monitored in positive mode. The MS parameters were used as follow: 500 °C TIS temperature (TEM); 5500 V ionspray voltage; 30 psi curtain gas (CUR); 50 psi nebulizing gas (GS1) and TIS gas (GS2); 120 and 90 V declustering potential (DP) for midazolam and nomifensine (IS), respectively; 10 V entrance potential; 38 and 28 eV collision energy (CE) for midazolam and IS; 15 V collision cell exit potential. The multiple reactions monitoring (MRM) transitions were *m*/*z* 326.2→291.1 for midazolam and *m*/*z* 239.2→91.0 for IS, respectively. For in vivo metabolism study, samples analyses were performed on the same LC-MS/MS platform. Separation was achieved on the Ultimate XB-C18 column (100 × 2.1 mm, 1.7 μm, Welch, Shanghai, China), and its temperature was maintained at 40 °C. Mobile Phase A was water containing 0.1% formic acid and Mobile Phase B was acetonitrile. The optimized gradient condition was as follows: 15% B from 0 to 2.0 min; 15–30% B from 2.0 to 3.0 min; 30–45% B from 3.0 to 10.0 min; 45–60% B from 10.0 to 25.0 min; 60–95% B from 25.0 to 28.0 min; 95% B from 28.0 to 32.0 min; 95–15% B from 32.0 to 32.1 min; and 15% B from 32.0 to 35.0 min. The flow rate was 0.3 mL/min. The following MS parameters were used: 600 °C TEM; −4500 V ionspray voltage; 30 psi CUR; 50 psi GS1 and GS2; −200 V DP. In accordance with an information dependent acquisition (IDA) procedure, an enhanced product ion (EPI) scan was triggered by an enhanced mass spectrum (EMS) and neutral loss (NL) scan, respectively, to characterize the metabolic products. The EMS scan range was from *m*/*z* 100 to 1300. EPI was triggered by IDA with a threshold of 10,000 cps, −70 eV CE, and 15 eV collision energy spread (CES). The NL of 162 and 132 Da were introduced as the survey scan. The NL scan range was from *m*/*z* 500 to 1300 with a 1000 cps NL–IDA–EPI threshold. As mentioned above, the same CE and CES were used in NL–IDA–EPI mode.

### 2.4. Data Analysis

Data analysis was carried out by Analyst software (version 1.6.1, AB SCIEX, Concord, ON, Canada). To calculate the percentage of parent compound remaining, average peak area ratio of analyte/IS of each sample was divided by that of the time zero sample. By plotting semi-logarithmic percentage of parent compound remaining versus incubation time, elimination rate constant (k) was calculated by the slope of linear regression of initial time points wherein log-linearity was observed. Half life (t_1/2_) was calculated by 0.693/k. Metabolites were identified and characterized by Peak View Software^TM^ 1.2 (AB SCIEX, Concord, ON, Canada).

## 3. Results and Discussion

### 3.1. Metabolic Stability

**Figure 1:**
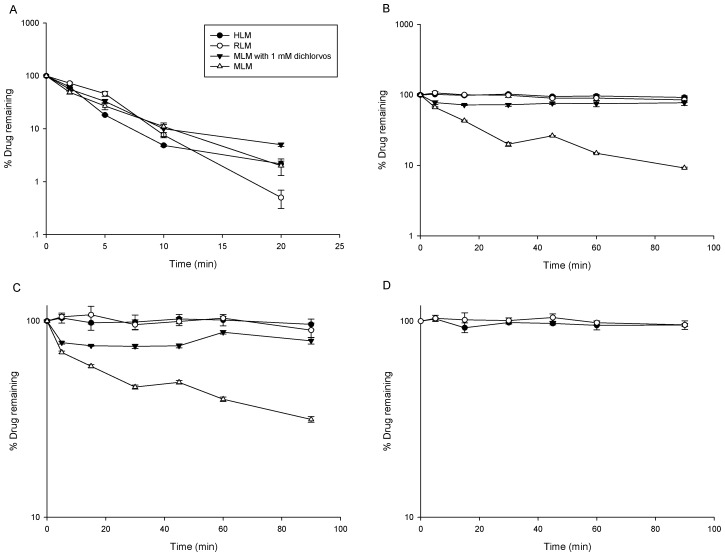
Metabolic stability of midazolam (**A**), Capilliposide B (LC-B) (**B**) and Capilliposide C (LC-C) (**C**) in human liver microsomes (HLM), rat liver microsomes (RLM), mouse liver microsomes (MLM), and MLM with diclorvos, respectively (*n* = 3). Metabolic stability of LC-A (**D**) in HLM and RLM (*n* = 3).

The positive control compound midazolam was rapidly metabolized by liver microsomes with t_1/2_ of 2.35, 3.85, and 3.73 min in rat liver microsomes (RLM), mouse liver microsomes (MLM), and human liver microsomes (HLM), respectively (Figure 1A). This demonstrated that active microsomes and appropriate incubation conditions were used in this metabolic stability study. As shown in Figure 1B–D, LC-B, LC-C, and LC-A were stable in RLM and HLM, but MLM could remarkably metabolize LC-B and LC-C with in vitro t_1/2_ of 27.7 and 63.0 min, indicating that the species difference in the metabolism of LC-B and LC-C was presented. Further, LC-B and LC-C underwent obvious degradation, regardless of the presence of NADPH. Thus, the metabolism of LC-B and LC-C was not NADPH-dependent in MLM. Recently, we employed a typical esterase inhibitor (dichlorvos) to prevent esterolysis of LC-B and LC-C in rat plasma [9,13]. In the current work, dichlorvos (1 mM) was added to the same incubation system, including MLM. As expected, the degradation of LC-B and LC-C was remarkably inhibited by dichlorvos (Figure 1), suggesting that esterase was the major metabolic enzyme for the biotransformation of LC-B and LC-C in MLM. Previously, Berry et al. reported that hepatic esterase activities varied between species [14]. As such, the species differences in the metabolism of LC-B and LC-C might be associated with altered hepatic esterase activities among liver microsomes from different species.

### 3.2. In Vivo Metabolism in Mice

#### 3.2.1. Metabolite Characterization by LC–QTRAP–MS/MS

**Figure 2:**
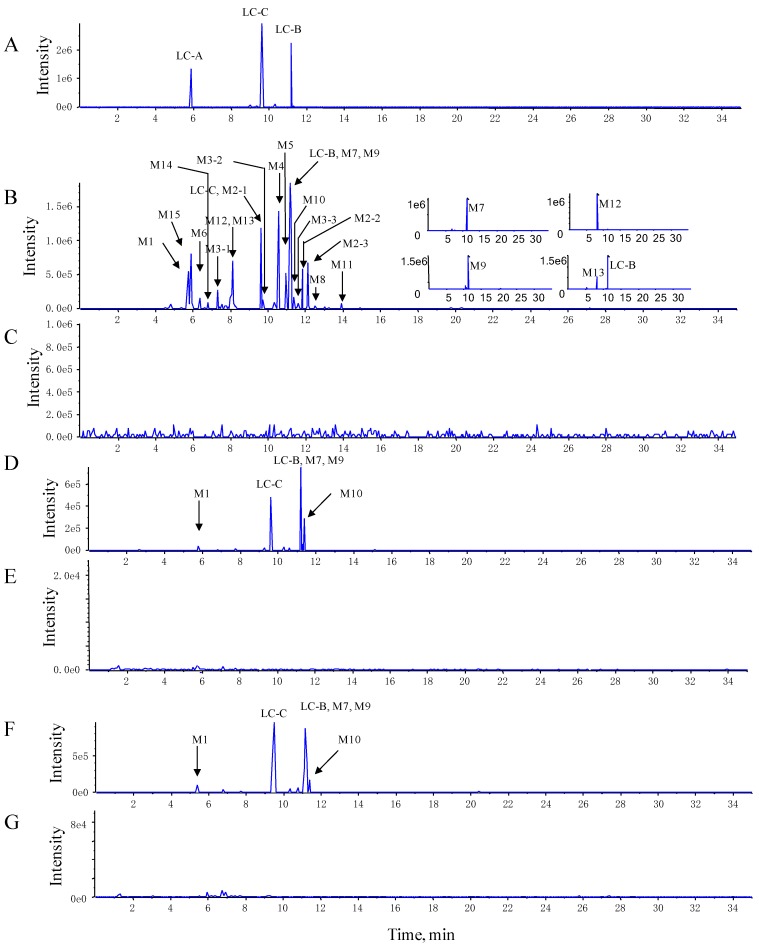
The representative extracted ion chromatograms (XIC) of LC-B, LC-C, and their metabolites. (**A**) The XIC of standard LC-B, LC-C, and LC-A. (**B**,**C**) The XIC of LC-B, LC-C, and their metabolites in pooled feces and blank feces. (**D**,**E**) The XIC of LC-B, LC-C, and their metabolites in pooled plasma and blank plasma. (**F**,**G**) The XIC of LC-B, LC-C, and their metabolites in pooled urine and blank urine.

**Figure 3:**
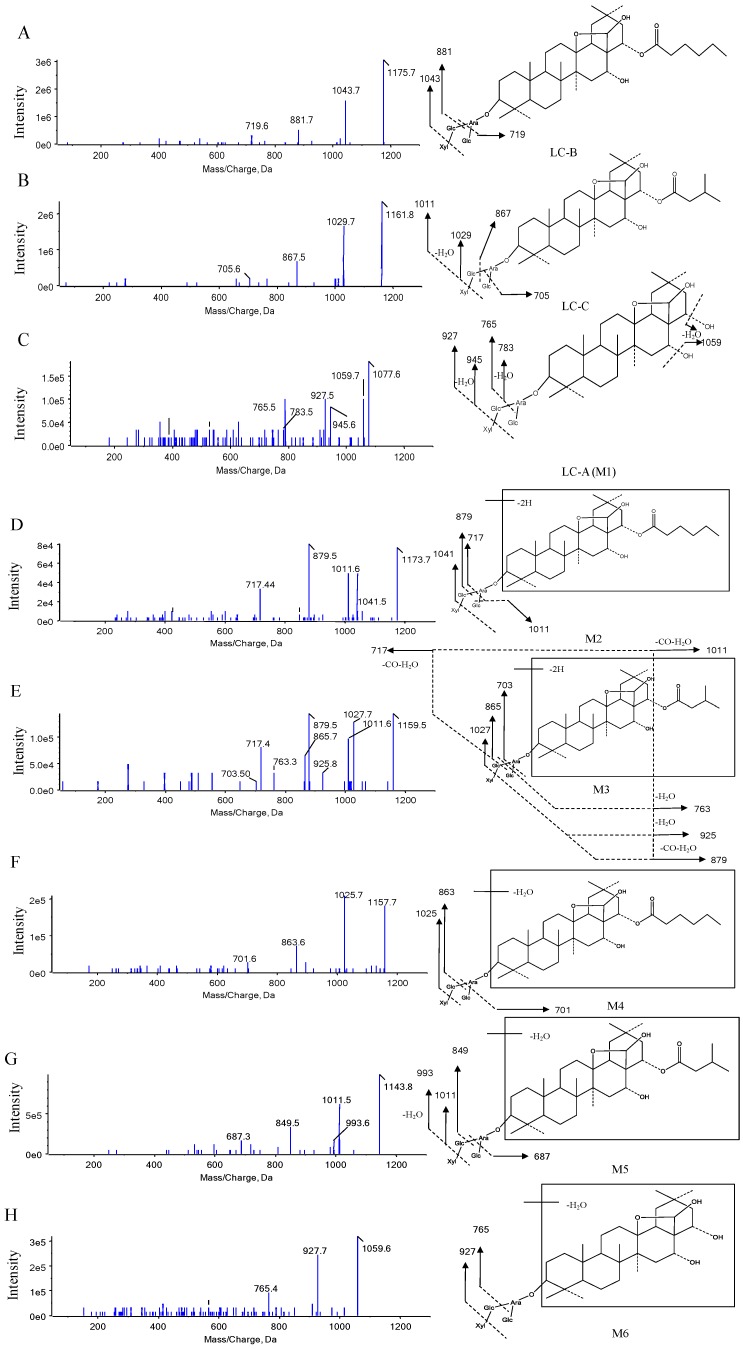
Typical MS/MS spectra of LC-B (**A**), LC-C (**B**), LC-A (**C**), M2-M6 (**D**–**H**), respectively.

To gain MS sensitivity, LC–QTRAP–MS/MS was optimized by standards of LC-B and LC-C. The MS/MS fragmentation behavior of LC-B and LC-C was helpful to deduce their respective metabolic products. In positive mode, the protonated molecule ions were obtained for LC-B and LC-C, but relatively stronger MS intensity together with a larger number of fragments were detected in negative ionization mode. The optimal collision energy was applied to generate more MS/MS fragmental information. Among different survey scan triggered MS/MS approaches, the NL–IDA–EPI method demonstrated greater versatility for conjugated metabolites [15,16,17]. According to the MS fragment ions of LC-B, LC-C, and their metabolites, the neutral losses of sugar moieties often occurred [12]. Thus, NL–IDA–EPI and EMS–IDA–EPI assays were simultaneously employed to identify and characterize their metabolites. In this study, 19 metabolites of LC-B and LC-C were detected and characterized in mice (Table 1, Figure 2 and Figure 3).

The LC-B, LC-C, and M1 (LC-A) were identified and confirmed according to product ion spectra and retention time of their standards (Figure 2A and Figure 3A–C). The [M-H]^−^ ions of LC-B, LC-C, and LC-A (M1) were observed at *m*/*z* 1175.7, 1161.8, and 1077.6, respectively (Figure 3A–C). Their major fragments resulted from neutral loss of sugar moieties and H_2_O. The [M-H]^−^ ion at *m*/*z* 1175.7 fragmented into *m*/*z* 1043.7, 881.7, and 719.6, respectively (Figure 3A). It was 132 Da higher than its product ion at *m*/*z* 1043.7, which indicated the loss of one molecular of xylose (Xyl). The product ion at *m*/*z* 881.7 originated from neutral loss of one molecular xylose and glucose (Glc). It further lost 162 Da (Glc) and turned into *m*/*z* 719.6. The [M-H]^−^ ion of *m*/*z* 1161.8 generated the product ions at *m*/*z* 1029.7, 1011.5, 867.5, and 705.6 (Figure 3B). The product ions at *m*/*z* 1029.7 and 1011.5 were generated by losing a xylose and xylose–H_2_O, respectively. The [M-H]^−^ of *m*/*z* 1161.8 fragmented into product ions at *m*/*z* 867.5 and 705.6, indicating Xyl-Glc and 2Glc-Xyl neutral loss. Interestingly, the largest number of metabolites was identified in pooled feces, compared to plasma and urine samples. LC-A (M1) was detected in all three matrices of interest. Figure 3C showed the MS/MS spectra of LC-A (M1). The product ions at *m*/*z* 1059.7 and 945.6 were 18 Da (H_2_O) and 132 Da (Xyl) less than its [M-H]^−^ ion at *m*/*z* 1077.6, respectively. They further lost one molecular Xyl and Glc and turned into *m*/*z* 927.5 and 783.5, respectively. Fragment ion at *m*/*z* 783.5 was 18 Da (H_2_O) higher than that at *m*/*z* 765.5.

The M2, M3, and M15 formed [M-H]^−^ ions at *m*/*z* 1173.7, 1159.5, and 1075.6, respectively (Figure 2B, Figure 3D,E and Figure S1). These seven metabolites were only detected in pooled feces (Figure 2B,D,F). Based on 2 mass unit difference of [M-H]^−^ ions from LC-B, LC-C, and LC-A, M15, M2, and M3 were assigned as their corresponding dehydrogenated products. However, the position of such a metabolic process remained unknown due to limited information of fragmentation pattern. The fragment ions of M2, M3, and M15 at *m*/*z* 1041.5 (-Xyl), 1011.6 (-Glc), 879.5 (-Xyl-Glc), and 717.4 (-Xyl-2Glc), *m*/*z* 1027.6 (-Xyl), 1011.7 (-C_5_H_10_O_2_-CO-H_2_O), 925.8 (-Xyl-C_5_H_10_O_2_-H_2_O), 879.5 (-Xyl-C_5_H_10_O_2_-CO-H_2_O), 865.7 (-Xyl-Glc), 763.3 (-Xyl-Glc-C_5_H_10_O_2_), 717.4 (-Xyl-Glc-C_5_H_10_O_2_-CO-H_2_O), and 703.5 (-Xyl-2Glc), *m*/*z* 943.6 (-Xyl), 925.7 (-Xyl-H_2_O), 879.7 (-Xyl-2H_2_O-CO), 781.5 (-Xyl-Glc), and 763.4 (-Glc-Xyl-H_2_O) were founded in the MS/MS spectra, which indicated that CO, H_2_O, Xyl, and Glc were lost from their [M-H]^−^ ions (Figure 3D,E and Figure S1).

The M4, M5, and M6 were 18 Da less than LC-B, LC-C, and LC-A, respectively, suggesting the loss of H_2_O from their parents. The three dehydrated metabolites were also only detected in pooled feces rather than in plasma and urine samples (Figure 2B,D,F). Likewise, the position of dehydration remained unclear, because their parents were multiple hydroxyl-containing compounds. Their product ions underwent a serial of neutral loss of sugar moieties, that is, the fragment ions of M4, M5, and M6 at *m*/*z* 1025.7 (-Xyl), 863.6 (-Xyl-Glc), 701.6 (-Xyl-2Glc), and *m*/*z* 1011.5 (-Xyl), 993.6 (-Xyl-H_2_O), 849.5 (-Xyl-Glc), 687.3 (-Xyl-2Glc), and *m*/*z* 927.7 (-Xyl), 765.4 (-Xyl-Glc) (Figure 3F–H).

The M7 and M8 demonstrated [M-H]^−^ ions at *m*/*z* 1043.1 and 1013.6, respectively (Figure S1). These results indicated that one xylose or glucose was lost from LC-B, respectively. M7 was found in all three matrices of interest, while M8 was only detected in pooled feces (Figure 2B,D,F). The mass spectra of M7 and M8 showed the same product ions at *m*/*z* 881.3 and 719.2 after losing xylose or glucose, which resembled the mass behavior of the parent compound.

The M9 gave [M-H]^−^ ion at *m*/*z* 1029.5 (Figure S1). It was assigned as a deglycosylated product of LC-C *via* losing xyl. Its major product ions at *m*/*z* 867.6 and 705.4 were the same as those of LC-C (Figure S1). Similarly, M10 (*m*/*z* 999.6) was 162 Da less than LC-C. M11 (*m*/*z* 867.5) lost Glc-Xyl from its parent compound (Figure S1). Thus, they were characterized as deglycosylated products of LC-C.

The M12 presented [M-H]^−^ ion at *m*/*z* 1177.7 and main fragment ions at *m*/*z* 1045.7 and 883.4. Its [M-H]^−^ ion and fragmental ions were 16 mass units larger than that of LC-C. Therefore, M12 was proposed to be a hydroxylated metabolite, which was only detected in pooled feces, after inserting an oxygen atom into its parent compound (Figure 2B and Figure S1).

The M13 and LC-B demonstrated the same [M-H]^−^ ion at *m*/*z* 1175.7, but it was 14 Da larger than that of the protonated LC-C. M13 was only found in feces and its retention time was found to be 8.13 min (Figure 2B). According to the mass difference from the parent compound, M13 was produced after either introduction of one oxygen atom with dehydrogenation or methylation. The main fragmental ions at *m*/*z* 1043.7 (-Xyl), 881.7 (-Xyl-Glc), and 719.6 (-Xyl-2Glc) via the neutral loss of sugar moieties (Figure S1). M14 was only found in feces and its retention time was 7.36 min (Figure 2B). The [M14-H]^−^ ion was 16 mass units larger than that of LC-B, suggesting that hydroxylation occurred in the skeletal structure of LC-B. It fragmented into *m*/*z* 1059.8, 897.6, and 735.5, which indicated xylose, Xyl-Glc, and Xyl-2Glc were lost from its deprotonated molecular ion (Figure S1).

#### 3.2.2. Proposed Metabolic Pathway of LC-B and LC-C in Mice

**Figure 4:**
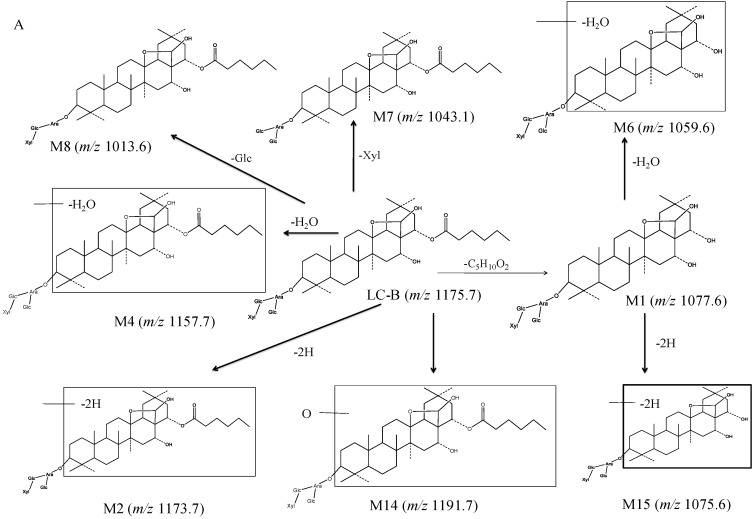

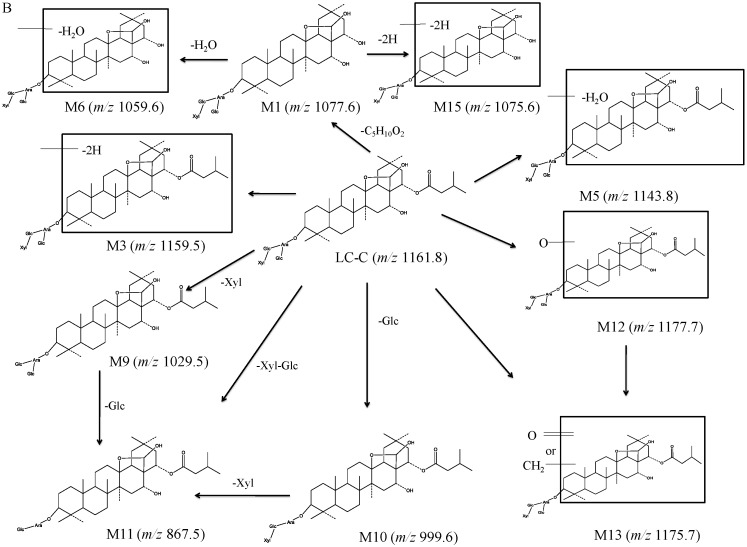
The proposed metabolic pathway of LC-B (**A**) and LC-C (**B**) in mice following oral dosing of total saponins of *L. capillipes* Hemsl extract (LCE).

To facilitate understanding of the systemic exposure of LC-B and LC-C, metabolic study was conducted in mice. A total of 19 metabolites were characterized. Figure 4 shows the proposed metabolic pathway. LC-B and LC-C mainly underwent deglycosylation, dehydrogenation, dehydration, esterolysis, and hydroxylation processes. Esterlysis and deglycosylation were found to be the major metabolic pathway in mouse plasma and urine. In this study, all of 19 metabolites were found in feces, while only four metabolites, LC-A (M1), M7, M9, and M10, were detected in all three matrices of interest (Table 1). LC-A was an esterolytic product of LC-B and LC-C. After oral administration of LCE, LC-B and LC-C could be hydrolyzed by intestinal microflora. On the other hand, the absorbed LC-B and LC-C could be metabolized by esterase in vivo. Then, LC-A was excreted from urine and feces, respectively. M1 was detected in all of three matrices follow oral administration in the present study, which was in good agreement with our previous report [9,11]. M7, M9 and M10 originated from LC-B or LC-C by losing one molecular of saccharide moiety. M9 (*m*/*z* 1029.5) has been identified as a main metabolite of LC-C, produced by intestinal microflora [12]. These results suggested that the terminal groups of saccharide moieties in LC-B and LC-C were readily lost both *in vivo and in vitro*. M7, M9, and M10, as major metabolites of their parent, were produced in intestine and entered systemic circulation, followed by excretion from urine and feces. Possibly, it was easy to detect such metabolites with relatively larger amounts in vivo. LC-A (M1), M6, and M15 might be derived from both parent compounds, while other metabolites were generated from either LC-B or LC-C. Previously, we reported that 92.7% of LC-C could be metabolized in intestinal microflora [12]. Subsequently, six metabolites were characterized as deglycosylation and/or esterolysis products [12]. Based on previous study, the intestine might be the major metabolic site of LC-B and LC-C. LC-A (M1) was mainly distributed in mouse liver after intravenous dosing of LC-B [10]. The current metabolic stability investigation demonstrated that LC-B and LC-C were subject to hydrolysis by esterase in MLM in vitro. These results indicated that esterases mediated hydrolysis could be major metabolic pathway of LC-B and LC-C in mice. Taken together, intestinal microflora mediated deglycosylation/hydrolysis together with subsequent hepatic esterolysis represent a significant proportion of the biotranformation process for LC-B and LC-C in mice. Additionally, dehydration, dehydrogenation, and hydroxylation may contribute, at least in part, to the metabolic clearance of LC-B and LC-C in mice.

In a previous study, LCE demonstrated significant antitumor activities following oral administration in nude mice [7]. The metabolic products of LC-C generated in intestinal microflora also demonstrated a strong anticancer effect in vitro [12]. Moreover, LC-A, a metabolite of LC-B and LC-C, exhibited a significant cytotoxicity against HepG2 cell lines [12]. In the present study, two parent compounds together with several previously reported metabolic products of LC-C produced in intestinal microflora were detected and characterized in mouse plasma. These metabolites are likely to be related with antitumor pharmacological efficacy. Future study will be required to unambiguously investigate the pharmacological activity of such bioactive metabolites in vivo.

## 4. Conclusions

In this study, the metabolic stability of LC-A, LC-B, and LC-C in HLM, MLM, and RLM was investigated. The results showed that LC-B and LC-C were stable in HLM and RLM, but not in MLM. Their metabolic stability could be improved by inhibiting esterolysis using dichlorvos. In addition, this is the first report concerning metabolism of LC-B and LC-C in vivo. Nineteen metabolites were characterized in mice. A number of biotransformation reactions, including deglycosylation, dehydrogenation, dehydration, esterolysis, and hydroxylation were involved. Particularly, deglycosylation and esterolysis could be the primary biotransformation reactions. Thereafter, the metabolic pathway of LC-B and LC-C in mice following oral dosing of LCE was proposed. According to the current and previous findings, intestinal microflora mediated deglycosylation/hydrolysis together with subsequent hepatic esterolysis played a critical role in the metabolic clearance of LC-B and LC-C in mice.

## Figures and Tables

**Table 1:** Characterization of the metabolites of Capilliposide B (LC-B) and Capilliposide C (LC-C) in mice by LC–QTRAP–MS/MS.

Name	Metabolic Description	Formula	Retention Time (min)	[M-H]^−^	Fragment Ions	Mouse		
						Plasma	Urine	Feces
LC-B	Parent	C_58_H_96_O_24_	11.20	1175.7	1043.7, 881.7, 719.6	×	×	×
LC-C	Parent	C_57_H_94_O_20_	9.65	1161.8	1029.7, 1011.5, 867.5, 705.6	×	×	×
M1 (LC-A)	Esterolysis	C_52_H_86_O_23_	5.88	1077.6	1059.7, 945.6, 927.5, 783.5, 765.5	×	×	×
M2	LC-B dehydrogenation	C_58_H_94_O_24_	9.73, 11.86, 12.13	1173.7	1041.5, 1011.6, 879.5, 717.4	–	–	×
M3	LC-C dehydrogenation	C_57_H_92_O_20_	7.68, 9.75, 11.41	1159.5	1027.6, 1011.7, 925.8, 879.5, 865.7, 763.3, 717.4, 703.5	–	–	×
M4	LC-B dehydration	C_58_H_94_O_23_	10.57	1157.7	1025.7, 863.6, 701.6	–	−	×
M5	LC-C dehydration	C_57_H_92_O_23_	10.98	1143.8	1011.5, 993.6, 849.5, 687.3	–	–	×
M6	LC-A dehydration	C_52_H_84_O_22_	6.39	1059.6	927.7, 765.4	–	–	×
M7	LC-B deglycosylation of xylose	C_53_H_88_O_20_	11.20	1043.1	881.3, 719.2	×	×	×
M8	LC-B deglycosylation of glucose	C_52_H_86_O_19_	12.21	1013.6	881.7, 719.4	–	–	×
M9	LC-C deglycosylation of xylose	C_52_H_86_O_20_	11.24	1029.5	867.5, 705.4	×	×	×
M10	LC-C deglycosylation of glucose	C_51_H_84_O_19_	11.39	999.6	897.6, 867.6, 765.4, 705.5, 659.5	×	×	×
M11	LC-C deglycosylation of glucose and xylose	C_46_H_76_O_15_	13.94	867.5	705.4	–	–	×
M12	LC-C hydroxylation	C_58_H_98_O_24_	8.18	1177.7	1045.7, 1027.8, 883.4	–	–	×
M13	LC-C methylation or hydroxylation with dehydrogenation	C_58_H_96_O_24_	8.13	1175.7	1043.7, 881.7, 719.6	–	–	×
M14	LC-B hydroxylation	C_57_H_92_O_26_	7.36	1191.7	1059.7, 897.7, 735.4	–	–	×
M15	LC-A dehydrogenation	C_52_H_84_O_23_	5.91	1075.6	943.6, 925.7, 879.7, 781.5, 763.4	–	–	×

## Supplementary Materials

The following are available online at http://www.mdpi.com/1999-4923/10/4/178/s1, Figure S1: The typical MS/MS spectra of M7 (**A**), M8 (**B**), M9 (**C**), M10 (**D**), M11 (**E**), M12 (**F**), M13(**G**), M14(**H**) and M15(**I**).
