# Supplementary material for: Metabolic Stability and Metabolite Characterization of Capilliposide B and Capilliposide C by LC–QTRAP–MS/MS
Source: Pharmaceutics. 2018 Oct 8;10(4):178. doi: 10.3390/pharmaceutics10040178 (PMC6321230; doi:10.3390/pharmaceutics10040178)
Supplement: Supplementary file 1 [file pharmaceutics-10-00178-s001.pdf]

pharmaceutics

MDPI

Supplementary Materials: Metabolic stability and characterization of Capilliposide B and Capilliposide C by LC-QTRAP-MS/MS

Zhongzhe Cheng, Xing Zhou, Zhifeng Du, Wenyi Li, Bingying Hu, Jingkui Tian, Lin Zhang, Jiangeng Huang and Hongliang Jiang

Figure S1. The typical MS/MS spectra of M7 (**A**), M8 (**B**), M9 (**C**), M10 (**D**), M11 (**E**), M12 (**F**), M13(**G**), M14(**H**) and M15(**I**).

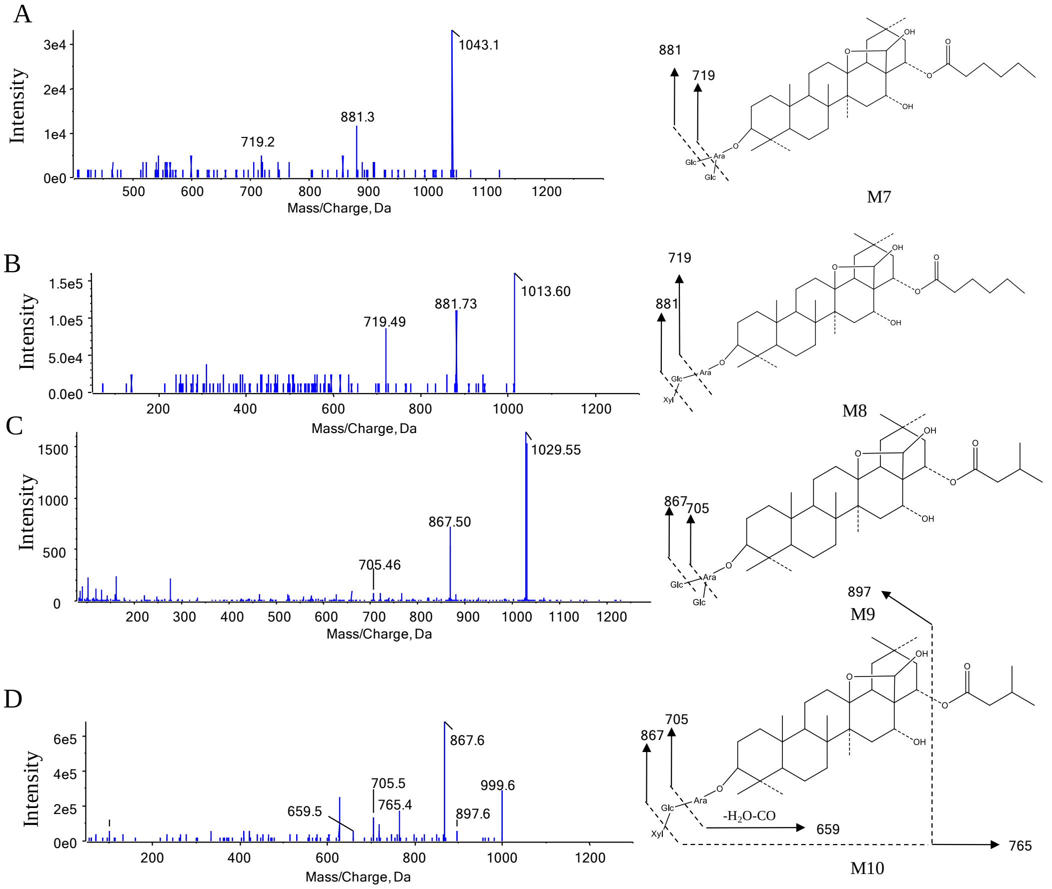

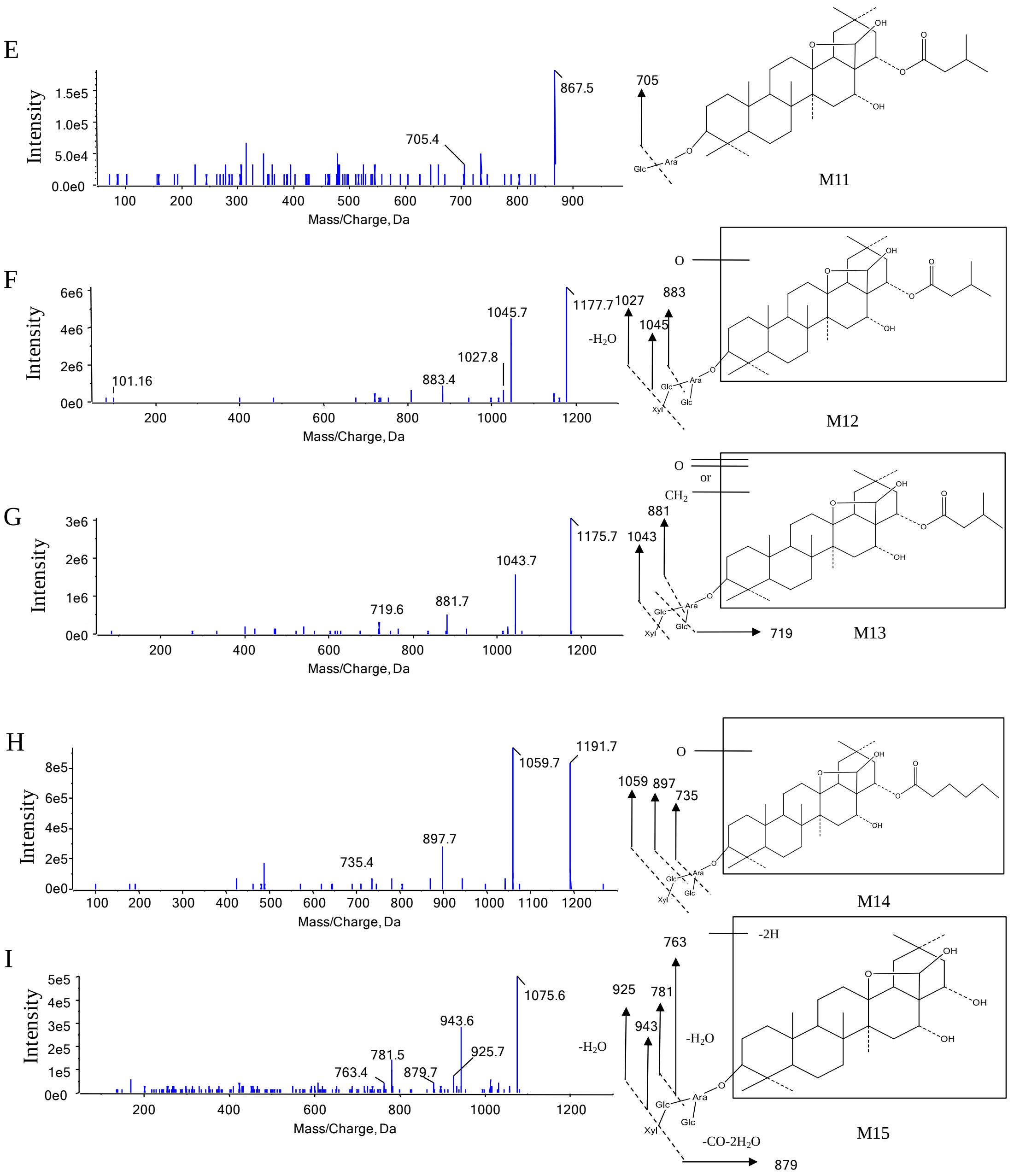

E
Intensity
1.5e5
1.0e5
5.0e4
0.0e0
867.5
705.4
Mass/Charge, Da
705
Glc
Ara
M11
F
6e6
4e6
2e6
0e0
101.16
883.4
1027.8
1045.7
1177.7
1027
883
1045
-H2O
O
Glc
Xyl
Ara
M12
G
3e6
2e6
1e6
0e0
719.6
881.7
1043.7
1175.7
O
or
CH2
881
1043
719
M13
H
8e5
6e5
4e5
2e5
0e0
735.4
897.7
1059.7
1191.7
1059
897
735
M14
I
5e5
4e5
3e5
2e5
1e5
0e0
763.4
781.5
879.7
925.7
943.6
1075.6
763
-2H
925
781
943
-H2O
-CO-2H2O
879
M15
